# COVID-19 pandemic impacts on global inland fisheries

**DOI:** 10.1073/pnas.2014016117

**Published:** 2020-11-02

**Authors:** Gretchen L. Stokes, Abigail J. Lynch, Benjamin S. Lowe, Simon Funge-Smith, John Valbo‐Jørgensen, Samuel J. Smidt

**Affiliations:** ^a^School of Natural Resources & Environment, University of Florida, Gainesville, FL 32611;; ^b^National Climate Adaptation Science Center, US Geological Survey, Reston, VA 20192;; ^c^Fisheries and Aquaculture Department, Food and Agriculture Organization of the United Nations, 00153 Rome, Italy;; ^d^Soil and Water Sciences Department, University of Florida, Gainesville, FL 32611

**Keywords:** coronavirus, freshwater fish, food security, livelihoods, recreation

## Abstract

The COVID-19 pandemic has led to environmental recovery in some ecosystems from a global “anthropause,” yet such evidence for natural resources with extraction or production value (e.g., fisheries) is limited. This brief report provides a data-driven global snapshot of expert-perceived impacts of COVID-19 on inland fisheries. We distributed an online survey assessing perceptions of inland fishery pressures in June and July 2020 to basin-level inland fishery experts (i.e., identified by the Food and Agriculture Organization of the United Nations across the global North and South); 437 respondents from 79 countries addressed 93 unique hydrological basins, accounting for 82.1% of global inland fish catch. Based on the responses analyzed against extrinsic fish catch and human development index data, pandemic impacts on inland fisheries 1) add gradation to the largely positive environmental narrative of the global pandemic and 2) identify that basins of higher provisioning value are perceived to experience greater fishery pressures but may have limited compensatory capacity to mitigate COVID-19 impacts along with negative pressures already present.

Inland fisheries are important global contributors to food security, livelihoods, and ecosystem services (1) while supporting a viable livelihood alternative and societal buffer with low barriers to entry (2) in rapid-onset crises (e.g., wars and pandemics). Preliminary evidence of the “anthropause” (3) from the COVID-19 pandemic has suggested some positive ecological responses and improved ecosystem functioning (4) from reduced human activity. However, the ecological responses of some provisioning resources may vary depending on how the resources are used (e.g., fish for food or leisure). Inland fisheries may offer short-term, midpandemic employment (e.g., fishing and processing), food resources, and socially distanced recreation, but sustained yield is also challenged by shifting harvest restrictions and consumer demands exacerbated or compounded by preexisting pressures (e.g., dams and pollution). This brief report assesses expert-perceived pressures from COVID-19 (increased, no change, or lessened) on global inland fisheries and relates these data to three use indicators: 1) reported inland fish catch, 2) human development index [HDI; composite metric: life expectancy, education, and living standard (5)], and 3) fishery provisioning value (high catch and/or low HDI).

## Results

We received 437 survey responses from 79 countries and 93 hydrological basins [HydroBASINS Level-3 (6)]. These basins account for 82.1% of reported global inland fish catch (7); 148 respondents (34%) perceived increased pressure from COVID-19, 161 (37%) perceived no change, and 128 (29%) perceived lessened pressure (*Materials and Methods*). The term “pressure” used here is broadly inclusive of multiple fishery-specific pressures (fishing, environmental, and other external drivers), as the greatest threats to inland fisheries originate from outside the fishing sector (8).

**Fig. 1.**
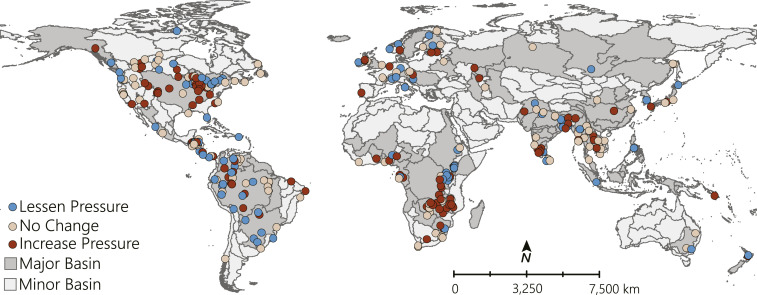
Perceived inland fisheries responses to COVID-19, where responses (*n* = 359, excludes nongeolocated responses) and major basins account for 82% and 95% of global inland fish catch, respectively.

Clear clusters of like responses emerged (Fig. 1). Notably, respondents perceived increased pressure in southeastern Asia and eastern Africa, lessened pressure in southeastern South America and Oceania, and highly mixed responses in North America and Europe. Pertinent individual respondent comments [by river-basin name (9)] help rationalize these clusters: 1) lessened pressure from initial fishing bans or restrictions (Murray–Darling and Orange), commercial fishing reductions (Ganges), and halted tourism (Lempa); 2) no change in pressure where increased domestic demands counteract transboundary trade restrictions (Lake Malawi), temporary restaurant and tourism closures negate long-term economic pressures (Guadiana), and fish harvest and sales continue (Santiago); and 3) increased pressure where high unemployment increases subsistence fish catch (Brahmaputra), fisheries provide livelihood safeguards from job losses (Mekong), and lost tourism livelihoods increase illegal fishing in nature reserves (San Juan).

**Fig. 2.**
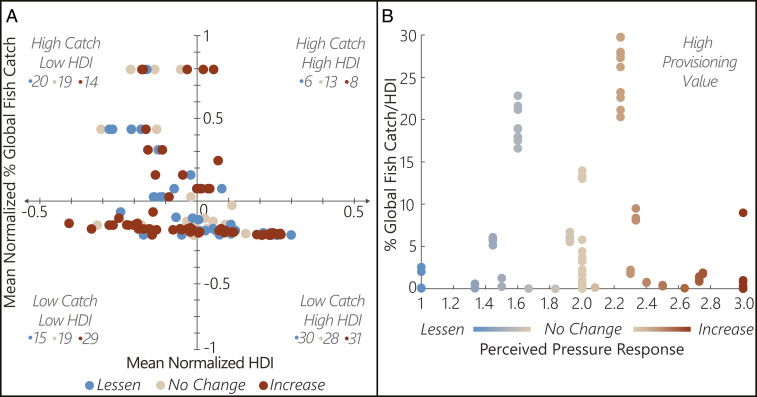
Perceived COVID-19 pressure responses (*n* = 232, excludes responses outside major basins, without HDI data, and nongeolocated) related to (*A*) mean normalized HDI and global fish catch and (*B*) provisioning ecosystem service value index (percent global inland fish catch/HDI), where high index values capture high catch and/or low HDI.

Across major basins (i.e., basins accounting for 95% of inland catch; *n* = 232, 50 basins), prominent patterns emerged: perceived increased pressure in areas of relatively lower catch and lower HDI (46.0%); decreased pressure in higher-catch, lower-HDI areas (38%); no change in higher-catch, higher-HDI areas (48%); and evenly distributed pressure responses (±4%) in lower-catch, higher-HDI areas (Fig. 2*A*). Catch–HDI quadrants represent 27, 23, 12, and 38% of responses, clockwise from lower left (Fig. 2*A*). Respondents perceived increased pressure to fisheries with notable provisioning ecosystem service value, measured by high production and/or subsistence role (i.e., low HDI) (Fig. 2*B*).

## Discussion

This study contributes a data-driven perspective to COVID-19 impacts on inland fisheries complementary to studies on fishers (10) and at-risk fishes (11). Responses applied geographically to a deductive pressure typology (i.e., three provided response choices) yield locally clustered perceptions of COVID-19 impacts. This distribution extending beyond positive evidence of direct (e.g., reduced human–wildlife disturbance) and indirect (e.g., reduced pollution) benefits of the “anthropause” reflects interdependent, and often essential, provisioning services of inland fisheries (e.g., food and income). Such may apply to other extracted multiuse resources (e.g., timber and cultivated crops).

### Perceived Impacts.

Respondent comments offer several important considerations for spatial patterns of fishery pressure, including 1) preexisting, persistent, or confounded environmental stressors; 2) fishery composition (e.g., species), type, and access; 3) consumer behavior; 4) fishery management/enforcement; and 5) regional employment opportunities. Possible sources of respondent variation include 1) virus and lockdown onset, severity, and extent; 2) institutional adaptive capacity; and 3) temporal interpretation of pressures.

Respondents indicated decreased fishery pressure may arise from reduced market demand and stay-at-home orders preventing travel to and within fishing areas. In some tropical regions where the crisis coincided with peak fishing season, reduced access and fishing effort may also increase brood fish survival and replenish fish stocks (12). No perceived change in pressure may result from families consuming rather than selling fish or unaffected fishing activity from modest lockdown measures’ negligible economic disruption. Increased pressure may occur where fish are a primary source of food or income, recreational fishing is incentivized and accessible, or illegal fishing transpires from relaxed enforcement. Increased pressure also appeared connected to a surge in subsistence fishers as jobless urban or migrant laborers return to rural areas seeking alternative livelihoods. First-time fishers lacking system knowledge are more likely to introduce destructive fishing practices or harvest at-risk species (11).

### Links to Fishery Provisioning Services.

Lessened pressure in higher-catch, lower-HDI areas is supported by decreased commercial fishing or preexisting institutional oversight; no change or increased pressure in higher-catch, higher-HDI areas by continued or increased commercial fishing or counteracting short- and long-term impacts; and greatest response uniformity in lower-catch, higher-HDI areas by shifting consumer demands or societal buffers absorbing rapid-onset shocks (Fig. 2*A*). Notably, a higher proportion of increased pressure responses in lower-catch, lower-HDI areas likely captures human migration for alternative food sources, livelihoods by unemployed workers during a global pandemic, or limited management capacity in increasingly exploited fisheries (Fig. 2*A*). Ninety-five percent of reported inland fish catch comes from the global South, including 45% from low-income, food-deficit countries where fishery provisioning services are highly valued (13). Many such regions are subject to pandemic-related extreme poverty (14). Here, increased pressure from provisional explorations captured a greater portion of low-income, food-deficit basins than those in affluent, developed countries. COVID-19 may exacerbate adverse fishery conditions in low-income areas where negative fish stock impacts extend beyond a pandemic recovery timeline.

## Conclusions

COVID-19 effects on inland fisheries reflect the complexity of the pandemic. Crisis mitigation drives short-term compensatory impacts. Economic recovery efforts compounded by preexisting ecological stressors dictate longer-term fishery impacts. Based on the results of this study, which help improve the global understanding of COVID-19 impacts on natural resource use, we can conclude the following:

1) Inland fisheries, as a provisioning ecosystem service, add gradation to the predominately positive narrative of COVID-19 environmental impacts (e.g., a greater proportion of fisheries were perceived to experience increased or no change in pressure than those experiencing decreased pressure).
2) Inland fisheries with higher provisioning value for upholding livelihoods and nutrition are perceived at higher risk of increased pressures due to COVID-19.

## Materials and Methods

We distributed an electronic survey to ∼1,900 fishery professionals (basin-level experts identified by the Food and Agriculture Organization of the United Nations [FAO]) assessing perceived inland fishery pressures. Source groups (InFish, FAO, and the American Fisheries Society) represented management, research, and other sectors approximately equally. We asked, “In light of the current COVID-19 pandemic, what impact do you predict it may have on your fishery?” with the following available responses: 1) lessened pressure, 2) no change in pressure, or 3) increased pressure. No temporal constraints were specified. Respondents self-identified a fishery of expertise, indicated its location (selected geographic coordinates and/or region/basin), and could provide comments. We collected responses from 16 June to 15 July 2020. The University of Florida Institutional Review Board approved anonymous data collection and informed consent waivers (IRB 202000533). Data are available through the Consortium of Universities for the Advancement of Hydrologic Science, Inc. HydroShare data repository (15). We summarized basin-level (6, 9) global fish catch data with FAO permission (7). We subset responses (Fig. 2 *A* and *B*) to quantify normalized mean fish catch and HDI (Fig. 2*A*) and basin-averaged responses to fish catch divided by HDI (Fig. 2*B*). See *SI Appendix* for extended methods.

## Supplementary Material

Supplementary File

## Data Availability

All scripts used for processing figures (Python file, MATLAB code), anonymized raw survey data (.csv file), and processed data files used in/created by the scripts (.xlsx files) have been deposited (COVID-19 Inland Fisheries Global Survey) in the Consortium of Universities for the Advancement of Hydrologic Science, Inc. (CUAHSI) HydroShare data repository (https://www.hydroshare.org/resource/43235c5188db4ae8a6589399d33c2efa/). Some study data are available upon request.
