# Supplementary material for: COVID-19 pandemic impacts on global inland fisheries
Source: Proc Natl Acad Sci U S A. 2020 Nov 2;117(47):29419–21. doi: 10.1073/pnas.2014016117 (PMC7703588; doi:10.1073/pnas.2014016117)
Supplement: Supplementary File [file pnas.2014016117.sapp.pdf]

PNAS
www.pnas.org

Supplementary Information for

COVID-19 pandemic impacts on global inland fisheries

Gretchen L. Stokes, Abigail J. Lynch, Benjamin S. Lowe, Simon Funge-Smith, John Valbo-Jørgensen, & Samuel J. Smidt

Gretchen Stokes
Email: gstokes@ufl.edu

This PDF file includes:

Supplementary text

www.pnas.org/cgi/doi/10.1073/pnas.2014016117

Supplementary Information Text

Extended Methods

We distributed a Qualtrics survey via anonymous link (initial and follow-up email distributions) to ~1900 fisheries professionals, including: American Fisheries Society members (~1250; Fish Habitat, Canadian Aquatic Resources, and International Fisheries Sections), FAO affiliates and collaborators (~500), and InFish network members (~150). Distribution included professionals in commercial, subsistence, and recreational fisheries and was not limited to fishery professionals from any one type of inland fishery. Additional snowball sampling was permitted. Surveys were available in English, Spanish, Portuguese, French, Korean, and Chinese. Respondents indicated their self-identified fishery (basin) of expertise location by one or both of the following: 1) pin drop inside their fishery's water body using a *Google Maps* extension (recorded as geographic coordinates) and/or 2) selecting their basin and/or region name from a provided list.

All responses were summarized and spatially examined by pressure response. Only responses with geographic coordinates (n=359; excluding those with basin/region name only (n=78)) are shown in Figure 1. We subset response data (n=232) (representing the original ratios of pressure responses within 3%) for Figure 2 analyses, where responses with basin/region names only (n=78), no HDI data (n=10), and outside major basins (n=117) were excluded. We omitted imaging three outlier points available in the referenced open-access dataset (2 in Figure 2a, 1 in Figure 2b). We included only comments pertinent to COVID-19 impacts on fisheries as qualitative results. We paraphrased comments by basin name to maintain respondent anonymity. Data were processed using Python (Version 2.7), MATLAB (Version R2018a), and ArcGIS (Version 10.6.1).
